# Genetic Structure, Function, and Evolution of Capsule Biosynthesis Loci in *Vibrio parahaemolyticus*

**DOI:** 10.3389/fmicb.2020.546150

**Published:** 2021-01-11

**Authors:** Shengzhe Bian, Wenhong Zeng, Qiwen Li, Yinghui Li, Nai-Kei Wong, Min Jiang, Le Zuo, Qinghua Hu, Liqiang Li

**Affiliations:** ^1^BGI Education Center, University of Chinese Academy of Sciences, Shenzhen, China; ^2^BGI-Shenzhen, Shenzhen, China; ^3^Shenzhen Key Laboratory of Unknown Pathogen Identification, Shenzhen, China; ^4^Jiangxi University of Traditional Chinese Medicine, Nanchang, China; ^5^Shenzhen Center for Disease Control and Prevention, Shenzhen, China; ^6^National Clinical Research Center for Infectious Diseases, Shenzhen Third People’s Hospital, The Second Hospital Affiliated to Southern University of Science and Technology, Shenzhen, China; ^7^School of Public Health (Shenzhen), Sun Yat-sen University, Guangzhou, China

**Keywords:** genetic structure, K antigen, capsule biosynthesis loci, *Vibiro parahaemolyticus*, serotype

## Abstract

Capsule-forming extracellular polysaccharides are crucial for bacterial host colonization, invasion, immune evasion, and ultimately pathogenicity. Due to warming ocean waters and human encroachment of coastal ecosystems, *Vibrio parahaemolyticus* has emerged as a globally important foodborne enteropathogen implicated in acute gastroenteritis, wound infections, and septic shock. Conventionally, the antigenic properties of lipopolysaccharide (LPS, O antigen) and capsular polysaccharide (CPS, K antigen) have provided a basis for serotyping *V. parahaemolyticus*, whereas disclosure of genetic elements encoding 13 O-serogroups have allowed molecular serotyping methods to be developed. However, the genetic structure of CPS loci for 71 K-serogroups has remained unidentified, limiting progress in understanding its roles in *V. parahaemolyticus* pathophysiology. In this study, we identified and characterized the genetic structure and their evolutionary relationship of CPS loci of 40 K-serogroups through whole genome sequencing of 443 *V. parahaemolyticus* strains. We found a distinct pattern of CPS gene cluster across different K-serogroups and expanded its new 3′-border by identifying *glpX* as a key gene conserved across all K-serogroups. A total of 217 genes involved in CPS biosynthesis were annotated. Functional contents and genetic structure of the 40 K-serogroups were analyzed. Based on inferences from species trees and gene trees, we proposed an evolution model of the CPS gene clusters of 40 K-serogroups. Horizontal gene transfer by recombination from other *Vibrio* species, gene duplication is likely to play instrumental roles in the evolution of CPS in *V. parahaemolyticus.* This is the first time, to the best of our knowledge, that a large scale of CPS gene clusters of different K-serogroups in *V. parahaemolyticus* have been identified and characterized in evolutionary contexts. This work should help advance understanding on the variation of CPS in *V. parahaemolyticus* and provide a framework for developing diagnostically relevant serotyping methods.

## Introduction

*Vibrio parahaemolyticus*, a Gram-negative halophilic bacterium, is taxonomically a notable member of the genus *Vibrio* within the family *Vibrionaceae*. It prevails in estuarine, marine, and coastal areas (Su and Liu, 2007; Nelapati et al., 2012; Ceccarelli et al., 2013; Zhang and Orth, 2013), and is typically isolated in a free-swimming state. Significant motility is conferred by a single polar flagellum in *V. parahaemolyticus*, which is capable of sensing both biotic and abiotic surfaces including zooplankton, fish, and shellfish, via impeded rotation (Gode-Potratz et al., 2011). Because of climate change and anthropogenic degradation of coastal environments, *V. parahaemolyticus* is gaining notoriety as an enteropathogen in humans worldwide, where people depend on seafood as a major nutritional source. Clinically, it can cause three major diseases, namely, gastroenteritis, wound infections, and septicemia (Daniels et al., 2000). In general, thermostable direct hemolysin (TDH) and TDH-related hemolysin (TRH) are two major virulence factors of *V. parahaemolyticus* implicated in its pathogenicity (Wang et al., 2015).

In host–pathogen interactions, bacteria leverage several extracellular polysaccharides to colonize hosts and cause disease. Gram-negative bacteria produce lipopolysaccharide (LPS), which is an integral component of the outer leaflet of bacterial outer membrane consisting of three structural moieties: lipid A, core oligosaccharide (core), and O-specific polysaccharide or O antigen (OAg) (Nikaido, 2003; Valvano, 2011). An additional capsular polysaccharide (CPS) (K antigen) may also be expressed, which is considered a significant virulence factor as it can increase bacterial survival upon phagocytosis by eukaryotic cells (Spinosa et al., 2007; Zaragoza et al., 2008) and blunt efficacy of antibiotics (Llobet et al., 2008; Geisinger and Isberg, 2015). Importantly, capsule is also advantageous for bacterial persistence and adaptation to harsh environments through protection from physical and chemical stresses, without sacrificing efficiency in the transfer of genetic materials between cells (Rendueles et al., 2018).

As a Gram-negative bacterium, *V. parahaemolyticus* produces a number of different somatic (O) and capsular (K) antigens, which have been exploited as a primary basis of strain classification (Nair et al., 2007). Being a pathogenic bacterium of multi-serotypes, *V. parahaemolyticus* can be classified into 13 O serotypes and 71 K serotypes (Han et al., 2008). Progress on the variation and dissemination of those serotypes has gone through different stages in its research history. *V. parahaemolyticus* was first discovered by Tsunesaburo Fujino in 1950 as a causative agent of foodborne disease following a large outbreak in Japan, which recorded 272 patient cases with 20 deaths after consumption of *shirasu* (Fujino et al., 1953). Before 1996, no particular serotypes of *V. parahaemolyticus* were associated with outbreaks. However, in the same year, a major outbreak arose in Kolkata, India, later known as the first pandemic, which was caused by strains with increased virulence. More than half of the patient isolates were serotype O3:K6 (Nair et al., 2007). Subsequently, the pandemic serotypes disseminate widely and rapidly. Within a few months, pandemic O3:K6 strains were identified in neighboring Vietnam, Indonesia, Bangladesh, Laos, Japan, Korea, and Thailand (Nair et al., 2007). They have consistently been detected globally (including Africa, Europe, North America, and Latin America) for years afterward (Ansaruzzaman et al., 2005; Nair et al., 2007; Velazquez-Roman et al., 2014). A variety of serotype variants have emerged such as O4:K68, O1:K25, and O1:KUT. However, they have identical molecular characteristics similar to the pandemic O3:K6 and have thus been collectively referred to as sero-variant of pandemic O3:K6 strains (Chowdhury et al., 2000b). Until 2016, a total of 49 pandemic serotypes (including 30 K-serogroups without untypeable) isolates from 22 countries across four continents (Asia, Europe, America, and Africa) were identified. All of these serotypes were detected in clinical isolates. Notably, because of its large geographical span and population, China has the most abundant pandemic serotypes among the 22 affected countries, which reach up to 26 serotypes and 12 K serogroups without untypeable (Han et al., 2016).

Traditionally, serotyping assays use commercially available antisera to identify *V. parahaemolyticus* strains, but this approach is limited by high costs, complicated procedure, cross-reactivity, and even subjective interpretation (Twedt et al., 1972). In contrast, molecular methods targeting serotype-specific genes can circumvent these shortages with proven superiority in specificity and sensitivity in the identification of bacterial serotypes (Liu et al., 2008). For instance, molecular methods for 13 O-serogroup detection and identification for *V. parahaemolyticus* have been developed, based on specific genes of O-serogroup genetic determinants (OGDs) (Chen et al., 2012). However, the CPS gene cluster (CPSgc) that determines K-serogroup has not been reliably identified to this date. In fact, there is a paucity of knowledge on the location of CPSgc in the *V. parahaemolyticus* genome, which was controversial up until 2010. By Tn5 mutagenesis in an O4:K8 serotype *V. parahaemolyticus* strain LM5312 (also known as BB22OP), Guvener and McCarter (2003) identified and proposed a locus *cpsA*–*cpsK* (VPA1403–VPA1413 in *V. parahaemolyticus* RIMD2210633 strain) spanning 11 genes on chromosome II for capsular polysaccharide biosynthesis without experimental verification of its functional correlation with K antigen. Subsequently, through homologous alignment with core oligosaccharide loci (core OS) and O-side chain in *Vibrio cholera* and comparison of restriction fragment polymorphisms in different serotypes, Okura et al. (2008) suggested a different locus, putative O-side chain gene cluster, determinate for K antigen, specifically, VP0214–VP0238 (*gmhD*–*rjg* in *V. cholerae* O139 MO45) on chromosome I of *V. parahaemolyticus* RIMD2210633 strain, although experimental evidence was not furnished. Subsequently, Chen et al. (2010) investigated these putative K-antigen genetic determinants in a pandemic O3:K6 isolate and confirmed by gene deletion that VP0214–VP0238 determines K antigen specifically but not O-antigen. Although location of K-antigenic determinant has been proven in pandemic O3:K6, the genetic structure and function of the loci encoding other K antigens remain obscure. Previous studies have found that most prevalent clones of *V. parahaemolyticus* stemmed from pandemic O3:K6 via serotype conversion (Chowdhury et al., 2000a, b, 2004a,b; Nair et al., 2007), whereas pandemic *V. parahaemolyticus* serotypes have become more and more diverse since 1996 (Nair et al., 2007). Recently, Pang et al. (2019) identified and annotated the CPSgcs of 55 K-serogroups from whole-genome sequences by using *gmhD*–*rjg* as borders. To advance a better understanding of *V. parahaemolyticus* pandemics, it is imperative to clarify the evolutionary relationship and divergence mechanism of CPS loci across different *V. parahaemolyticus* K-serogroups.

In this study, we have identified CPS loci of 40 K-serogroups from 64 serotypes, which include 24 pandemic K-serogroups (covering 86% pandemic K-serogroups in the world and 92% pandemic K-serogroups in China) and 16 non-pandemic K-serogroups. Their genetic structure, function, and evolutionary relationship of these 40 K-serogroup CPS loci were investigated. This work provides a framework for analyzing frequent mutations in *V. parahaemolyticus* K antigens and developing molecular tools for reliable serotyping.

## Materials and Methods

### Bacterial Culture and Conventional Serotyping

**FIGURE 1:**
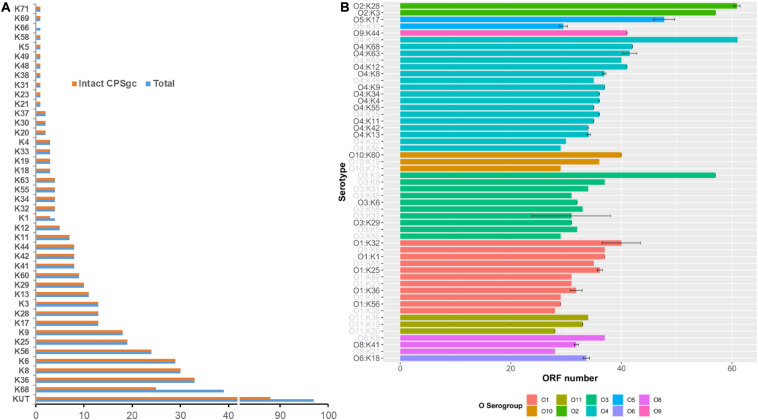
Summary of 443 *V. parahaemolyticus* strains by K-serogroups and coding capacity of *V. parahaemolyticus* CPS gene clusters. In panel **(A)**, pink bar indicates the number of strains for each K-serogroup used in this study, while the blue bar indicates the number of strains whose intact CPSgcs can be obtained. Panel **(B)** Indicates the coding capacity of CPS gene cluster of different serotypes. Bar graph shows the ORF number distribution of different serotypes CPS gene cluster without KUT/OUT, and these serotypes are grouped by O-serogroup with the same color. The error bars give SD for K serogroups which have more than one sample, and K-serogroups whose sample number is less than 3 are in gray font. The bars are ordered by mean ORF number of every O-serogroup.

A total of 443 strains of *V. parahaemolyticus* (Figure 1A and Supplementary Table 1) from sentinel hospitals were collected by Shenzhen CDC. *V. parahaemolyticus* were enriched in alkaline peptone water (pH 8.6; 3% NaCl) and incubated at 37° for 16 h on a shaker, then streaked onto *Vibrio* chromogenic agar incubated at 37° for 12 h for single colonies (Guangdong Huankai Microbial Science and Technology, Guangzhou, China). Potentially productive colonies were picked and streaked onto triple sugar iron slants (Guangdong Huankai Microbial Science and Technology, Guangzhou, China) and incubated at 37° for 16 h. They were then subjected to serotyping by serum slide agglutination tests using commercial antisera (Denka Seiken, Tokyo, Japan) according to the manufacturer’s protocol and the Chinese National Food Safety Standards: Food Microbiological Examination *Vibrio parahaemolyticus* Testing, GB 4789.7-2013.

### Whole-Genome Sequencing and Assembly

DNA extraction was performed using the Qiagen QIAamp DNA Mini Kit (QIAGEN, Germany) on the automatic nucleic acid extractor QIAGEN EZ1 Advanced XL (QIAGEN, Germany) according to the manufacturer’s instructions. Genomic DNA were sent for high-throughput sequencing by using the BGISEQ-500 platform (BGI-Shenzhen, China). After qualification checking using gel electrophoresis, DNA were fragmented and then processed by end repairing, A-tailing, adapter ligation, DNA size selection, circulation, and DNA nanoball formation according to the in-house SOP of library construction for BGISEQ-500. The DNA libraries with an insert size of 300 bp were sequenced using single-end 100 bp mode (SE100) or pair-end 100 bp mode (PE100) on a BGISEQ-500 sequencer. After quality trimmed using SOAPnuke (Chen et al., 2017), the rest of reads were assembled into contigs by Shovill^1^ with parameter depth = 0, minlen = 50.

### ORF Homologous Clustering, Annotation, and Gene Function Classification

*Function annotation*: Open reading frames (ORFs) of CPSgcs function were annotated by using prokka 1.13 (Seemann, 2014) and database selected from Swiss-Prot of UniProt. After this, if the ORFs cannot be annotated, they were blastn against the coding sequences of CPS loci of reference strain RIMD 2210633 (Chen et al., 2010) with E-value less than 1e-5, identity larger than 60%, and coverage larger than 60%.

*Selection of 40 representative K-serogroups*: To focus on comparisons between different K-serogroups, we selected 40 representative K-serogroup strains. First, we computed every strain’s K-antigen gene cluster length and ORF number. Then, for K-serogroups with more than two strains, the strains with K-gene cluster length and ORF number equal or similar to most strains in the K serogroup were chosen as reprehensive ones. For K-serogroups with less than three strains, we selected the representative strains at random.

*ORF homologous clustering and gene name assignment*: To make further research in ORF function, homologous ORFs of 40 representative strains were clustered by OrthoFinder (Emms and Kelly, 2019) with default parameters and assigned to orthogroups. Each orthogroup was designated as a gene group and was uniformly named as follows: count the ORF numbers for each gene name which is annotated as above using prokka, Swiss-Prot, or reference strain RIMD 2210633, and the annotated gene name with the largest proportion was chosen as the name of the orthogroup. In orthogroups where all ORFs could not be annotated as above, the orthogroup ID produced by the OrthoFinder program was assigned as their names.

*Gene function classification*: To clarify their functions in capsule biosynthesis pathway, genes annotated were assigned into four classes: pathway genes, processing and transportation genes, glycoltransferase genes, and function unknown or cannot be classified, following the procedure in Supplementary Figure 1. The annotation information of each ORF in 40 K-serogroup CPSgcs is listed in Supplementary Table 2.

### Sequence Alignment and Phylogenetic Analysis

Species tree of 40 K-serogroups was constructed based on whole lengths of CPS cluster using OrthoFinder v2.3.12 with default parameters to reveal the evolutionary relationship among the K-serogroups. Briefly, single-copy genes distributed in all K-serogroups were selected for genetic distance calculations, and gene tree construction was performed for one gene at a time by using Dendroblast (Kelly and Maini, 2013) implanted in OrthoFinder. Then, median distances of all closest distances across orthogroup gene trees were calculated as the distances between each species pair, which formed a single distance matrix. The species tree and branch lengths were inferred from this distance matrix by using FastME 2.1.5 (Lefort et al., 2015). Trees of homologous genes were generated by OrthoFinder v2.3.12 implanted with Dendroblast and FastME 2.1.5 in default settings.

Multiple sequence alignments of nucleotide sequences or protein sequences were done by MUSCLE v3.8.1551 (Edgar, 2004) with default parameters. Gene pairwise identity was calculated based on nucleotide sequence alignments. Phylogenetic trees based on protein sequence alignments were inferred by 1) using IQ-TREE (Nguyen et al., 2014) with the parameters -m GTR + F + R9, -bb 1000, -nt 8; 2) using FastTree v2.1.10 (Price et al., 2010) with a JTT + CAT model and doing 1,000 resamples for support value estimation; or 3) using raxml-ng v0.9.0 (Kozlov et al., 2019) with an LG + G4 model and 1,000 bootstrap replicates.

### Evolution Mechanism Analysis

Insertion events in 40 K-serogroup CPSgcs were identified through collinearity analysis among CPSgcs by using genoPlotR (Guy et al., 2010). Specifically, collinearity analysis among the 5′ and 3′ conserved flank regions was carried out to determine insertion genes potentially disrupting the conserved gene order in multi-CPSgcs. Collinearity analysis among the middle variable regions also revealed insertion genes with serial and conserved order among multi-CPSgcs. In addition, the source of potential insertion sequence was identified using blastn against GenBank with e-value equal to 0. Detection of recombination signals of CPSgcs was done by using RDP incorporating seven algorithms (Martin et al., 2015). Recombination events were taken as positive by verification in at least two algorithms (*p* value <0.05).

Generation mechanisms of multicopy genes were classified in two ways: If multicopy genes with global sequence homologs are located at the neighboring branches in a gene tree, the generation mechanism was taken as duplication. If multicopy genes have global sequence homologs but are located at different branches in a gene tree, then the generation mechanism was taken as recombination, suggesting certain copies of these multicopy genes were acquired by recombination from other donor sources.

## Results

### Discovery of Conserved 3′-Border Gene *glpX* of CPSgc

We selected 443 *V. parahaemolyticus* strains from different outbreaks during 2006–2017 from sentinel hospitals of Shenzhen, encompassing 71 serotypes and 40 K-serogroups, for whole genome sequencing. Draft genomes of good quality were obtained, with an average of 193 contigs and average 5.12 Mbp in total length for each genome. The average N50 length and average N90 length of assembled contigs are 335.44 and 59.48 kbp, respectively. Draft genomes of these 443 *V. parahaemolyticus* strains were then subjected to CPS loci gene cluster sequence extraction for subsequent analysis. Coding sequences (CDS) of every strain were first predicted by using prokka 1.13 (Seemann, 2014). Gene *gmhD* (VP0214 in reference strain RIMD 2210633) and *rjg* (VP0238 in RIMD 2210633) were chosen as 5′ border gene and 3′ border gene as previously reported (Chen et al., 2010). In addition, genes VP0215 and VP0237 (in RIMD 2210633), adjacent to the above two border genes, respectively, were considered as secondary border genes for more comprehensive extraction. For each strain, if a certain contig contains both a 5′-border gene (either *gmhD* or VP0215) and a 3′-border gene (either VP0237 or *rig*), then the putative whole-capsule gene clusters would be extracted from this contig. Border genes were queried by using blastn (Madden, 2013) with e-value less than 1e-5, identity larger than 60%, and coverage larger than 60%.

Following CDS prediction and annotation of obtained gene cluster sequences for putative CPS loci, we found that 5′-border gene *gmhD* is conserved in all strains, but the 3′-border gene *rig* and secondary 3′-border gene VP0237 are not fully conserved. Thus, we inferred that CPSgc of *V. parahaemolyticus* has a more conserved 3′-border gene. We check downstream ORFs along *rig* one by one and annotated them until no polysaccharide-related genes can be found. A polysaccharide-related gene *glpX* (VP0244 in RIMD 2210633), which is about five ORFs downstream of *rig*, was found distributing in all 443 strains including in the 418-well assembly CPSgcs. Therefore, we consider *glpX* to be a potential 3′-border gene and subject it to validation in subsequent analysis. Furthermore, the 3′ neighboring ORF of *glpX* is *zapB* (UniProt ID: P0AF3, coding cell division protein), which belongs to cell division–related gene cluster (data not shown). The genes between *glpX* and *rig* are polysaccharide-related genes: *glpX*, *tpiA*, and *hpcD*; more specifically, *tpiA* (402/418) and *hpcD* (398/418) also are highly conserved in all 418 well-assembled CPSgcs (including in 40 K-serogroup representative strains; see Table 1). In summary, *glpX* is an accurate 3′-border gene of CPSgc in *V. parahaemolyticus*. The following analyses are all based on the entire CPSgcs which are extracted by 5′-border gene *gmhD* and new 3′-border gene *glpX*.

**TABLE 1:** Sequence information on representative strains of 40 K-serogroups.

K-serogroup	Strain name*	Year of isolation	Length of CPSgc (bp)	ORF number of CPSgc	GenBank accession	CNSA accession**
K1	VP199	2009	37,916	37	MT898145	CNA0006925
K11	VP107	2007	35,823	35	MT898391	CNA0006832
K12	VP179	2012	41,196	41	MT898299	CNA0006903
K13	VP109	2008	34,798	34	MT898004	CNA0006834
K17	VP43	2009	57,948	50	MT898343	CNA0007181
K18	VP197	2015	32,885	34	MT898213	CNA0006923
K19	VP192	2007	37,137	33	MT898099	CNA0006918
K20	VP206	2008	29,547	28	MT898261	CNA0006934
K21	VP439	2009	29,690	28	MT898297	CNA0007180
K23	VP202	2008	36,002	31	MT898072	CNA0006930
K25	VP132	2008	36,845	36	MT898283	CNA0006860
K28	VP53	2010	67,564	61	MT898376	CNA0007199
K29	VP247	2008	31,094	31	MT898031	CNA0006976
K3	VP245	2008	64,678	57	MT898350	CNA0006974
K30	VP190	2008	32,416	29	MT898163	CNA0006916
K31	VP205	2008	34,720	34	MT898311	CNA0006933
K32	VP99	2010	46,096	42	MT898113	CNA0007243
K33	VP203	2008	32,308	29	MT898408	CNA0006931
K34	VP6	2008	38,125	36	MT898188	CNA0007217
K36	VP104	2010	33,497	32	MT898100	CNA0006830
K37	VP239	2009	27,236	26	MT898231	CNA0006969
K38	VP200	2008	28,581	28	MT898150	CNA0006928
K4	VP198	2015	37,080	36	MT898208	CNA0006924
K41	VP234	2010	32,326	32	MT898360	CNA0006964
K42	VP4	2008	35,037	34	MT898215	CNA0007195
K44	VP113	2008	42,006	41	MT898206	CNA0006839
K48	VP229	2008	34,007	31	MT898401	CNA0006958
K49	VP238	2010	39,254	35	MT898369	CNA0006968
K5	VP195	2008	30,811	32	MT898262	CNA0006921
K55	VP1	2008	37,003	35	MT898026	CNA0006927
K56	VP334	2016	29,389	29	MT898411	CNA0007068
K58	VP230	2012	33,084	33	MT898195	CNA0006960
K6	VP204	2017	33,084	32	MT898107	CNA0006932
K60	VP16	2015	43,197	40	MT898076	CNA0006893
K63	VP321	2008	43,707	42	MT898183	CNA0007054
K68	VP161	2010	44,725	42	MT898029	CNA0006887
K69	VP33	2011	36,120	31	MT898042	CNA0007074
K71	VP32	2012	31,101	29	MT898269	CNA0007063
K8	VP187	2011	39,442	37	MT898178	CNA0006912
K9	VP135	2008	39,047	37	MT898341	CNA0006863

* Representative strains selected from 443 strains. ** Besides deposited in GenBank, sequence assembly of the capsule biosynthesis loci was also deposited in the publicly available CNSA database (https://db.cngb.org/cnsa/) under project number CNP0000343.

### Coding Ability and Homologous Genes of CPSgc

The average length of 40 K-serogroup CPSgcs was found to be 37.84 kbp, and the average ORF number of 40 K-serogroup CPSgcs is 36. Correspondingly, ORFs are most abundant in K28 (61 ORFs) and least abundant in K38 (28 ORFs) (Figure 1B and Supplementary Table 1). Interestingly, the ORF number of CPSgc from O2:K28 and O2:K3 was apparently larger than that of other K-serogroups (Figure 1B) consistent with K28 and K3 that share a closer evolutionary relationship as found following evolution analysis in Section “Evolution and Groups of 40 K-Serogroup CPSgcs.”

To clarify coding structure and function of CPS loci, 40 CPSgcs from representative strains corresponding to 40 K-serogroups were selected for the following analysis (Table 1). Accordingly, 1,420 ORFs could be obtained from 40 K-serogroup representatives and were clustered into 219 homologous gene groups with 126 containing ≥2 gene members (Supplementary Table 2). This is slightly larger than that reported by Pang et al. (2019), possibly because of the expansion of newly identified borders. After annotation, these gene groups were subsequently grouped into four classes by general functions, including 48 pathway genes, 12 processing and transportation genes, 6 glycoltransferase genes, and 153 others (4 known functions but cannot be classified, and 149 unknown function) (Table 2 and Supplementary Table 2). The relative abundance of each gene class in the 40 K-serogroups is shown in Supplementary Figure 2.

**TABLE 2:** Gene number and identity within gene classes of 40 K-serogroup CPSgcs.

Frequency	Pathway genes		Processing and transportation genes		Glycoltransferase genes		Others		Total	
	Number	Mean MPD*	Number	Mean MPD	Number	Mean MPD	Number	Mean MPD	Number	Mean MPD*
100%	5	0.8388	1	0.7920	0	–	6	0.6968	12	0.7642
90–100%	0	–	1	0.3150	0	–	2	0.5785	3	0.4906
60–90%	2	0.3310	1	0.2390	2	0.1875	1	0.3090	6	0.2641
30–60%	10	0.3937	0	–	0	–	1	0.6310	11	0.4152
10–30%	13	0.2012	2	0.1960	1	0.197	9	0.6947	25	0.3783
<10%	18	0.3692	7	0.3437	3	0.2396	134	0.3324	162	0.3349
Total number/mean MPD*	48	0.3775	12	0.3456	6	0.2151	153	0.4238	–	–

* Average of mean pairwise identity (MPD) for genes in the corresponding category. – not applicable.

### Genetic Structural and Function Characteristics of CPSgc

To characterize the CPSgc further, we plotted the genetic structures of 40 K-serogroups according to their annotations. We found, in 40 K-serogroups, that the genes in the flank region are more conserved than those in the middle region (Figure 3). The genes at the flank region with distribution frequencies of more than 90% are designated as core genes: 12 of them have distribution frequencies of up to 100% (within-gene mean pairwise identity is between 55.4 and 97.4%). Another three genes have distribution frequencies of between 90 and 100% (VP0218, VP0219, *wzc*; within-gene mean pairwise identities are 48.8, 66.9, and 31.5%, respectively) (Figure 3 and Supplementary Table 2). Eight core genes in a conserved order, namely *gmhD*, VP0215, VP0216, *gfcB*, VP0218, VP0219, *kpsD*, and *wzc*, were sequentially located at the 5′ flank region in all K-serogroups except for K3 and K28 which only kept the previous four genes. There was only one short gene sparingly appearing between VP0218 and VP0219 in K56 and most K serogroups in group 5 (Figure 3). VP0215–VP0216–*gfcB* were designated as *yjbH*–*yjbG*–*yjbF*, and VP0219–*kpsD*–*wzc* were designated as *wbfE*–*wbfF*–*wzz* in Pang et al. (2019), both putatively implicated in CPS assembly (Chen et al., 2007.). This set of eight conserved genes, including *gmhD* reportedly lacking in K12 and K41 (Pang et al., 2019) due to incomplete sequencing, could be identified in our study. The other seven core genes, namely, *ugd*, *tpiA*, *hpcD*, OG011, OG012, OG013, and *glpX*, are sequentially distributed in the 3′ flank region. The intergenic regions between *ugd* and *tpiA*, and between *tpiA* and *hpcD* are frequently inserted by the gene(s) *rig* or/and *gtaB*, or long insert sequences (in K60, K68, K36, and K25), and by OG022 (Figure 3 and Supplementary Figure 3), suggesting high recombination activity.

**FIGURE 2:**
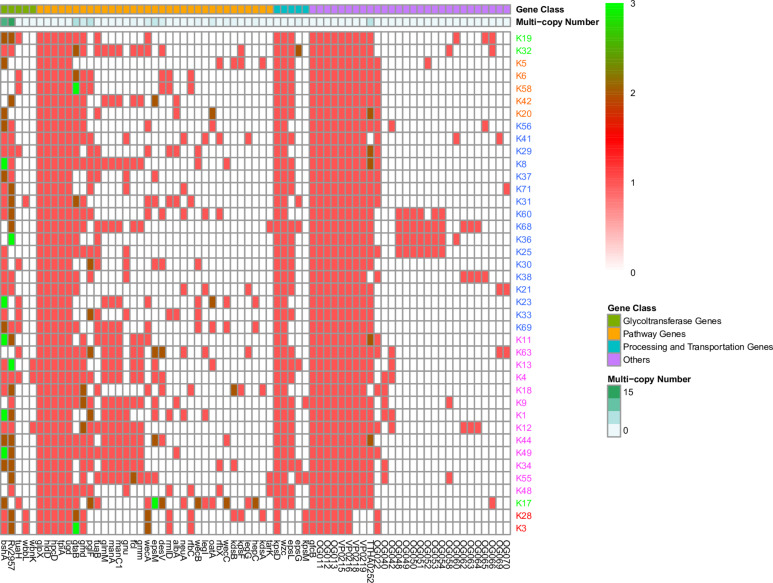
Gene presence or absence in 40 K-serogroup CPSgcs. Columns, corresponding to different genes, were ordered by gene class and occurring frequency in 40 K-serogroup reprehensive strains. In heatmap, each cell indicates the copy numbers of certain gene in corresponding K serogroup, and 1, 2, and 3 copies can be observed in different genes indicated in red, dark red, and green, respectively. Those genes existing in less than 2 K-serogroups were not included; for all gene versions, please refer to Supplementary Figure 4. K-serogroups were classified, ordered, and colored according to Figure 4 (red: group 1, green: group 2, orange: group 3, blue: group 4, purple: group 5).

**FIGURE 3:**
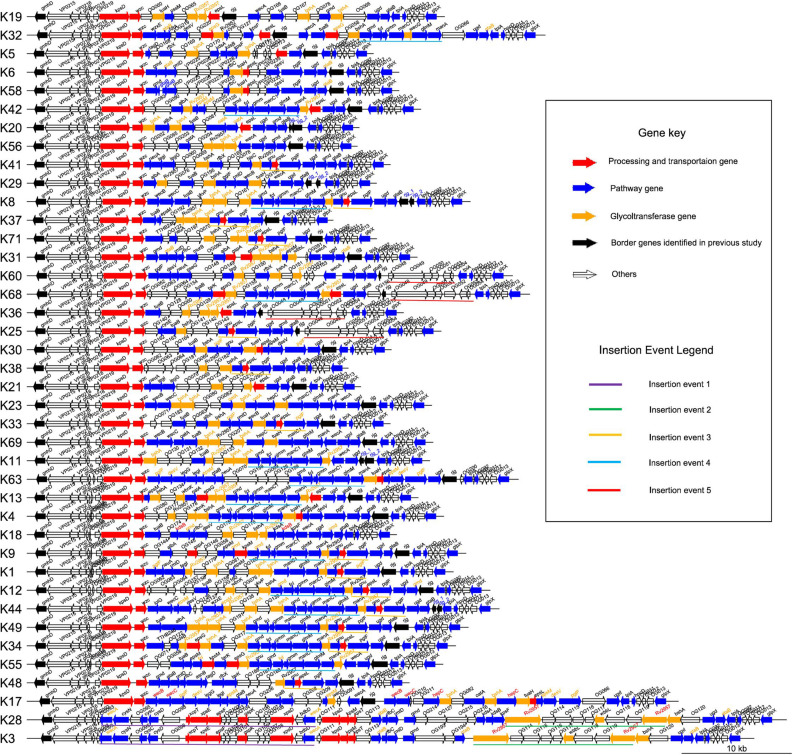
Genetic structure of 40 K-serogroup CPSgcs. Each line represents a gene cluster, and one arrow refers to one gene; the direction of arrow refers to the direction of genes. There are four filling colors of arrows representing four gene function classes: red refers to *processing and transportation genes*, blue refers to *pathway genes*, orange refers to *glycotransferase genes*, transparent refers to *others* (gene function unknown or cannot be classified), and black refers to two border genes *gmhD* and *rjg* identified in a previous study (Chen et al., 2010). In addition, gene names are in black font as default, whereas gene names in red font represent multi-copies originated by gene self-duplication; those in blue font represent multi-copies originated by nonsense mutation, and those in orange font represent multi-copies originated by recombination in corresponding K-serogroup. Underlines of five different colors highlight five insertion recombination events. K-serogroups were classified, ordered, and colored according to Figure 4 (red: group 1, green: group 2, orange: group 3, blue: group 4, purple: group 5).

**FIGURE 4:**
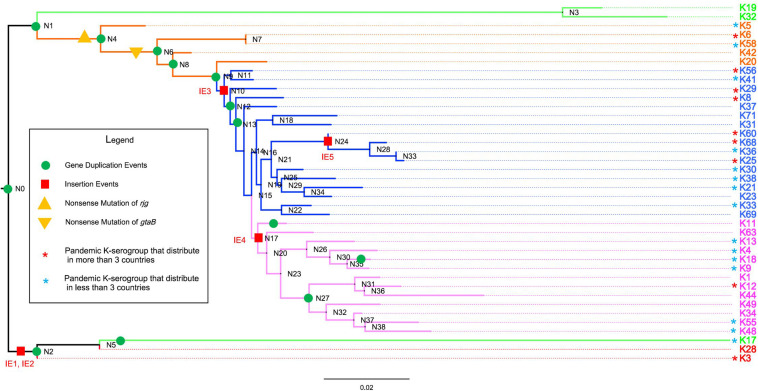
The evolution of 40 K-serogroup CPSgcs. Five groups of 40 K-serogroups are indicated by different colors for the branch and tips: group 1 in red, group 2 in green, group 3 in orange, group 4 in blue, and group 5 in purple. Node IDs were displayed, and the red square and green circle on certain branches refer to insertion events and duplication events, respectively. For gene duplication events, the concrete information is listed in Table 4. For insertion events, the sequence of IE1 (insertion event 1) contains *wecA*, *cysN*, OG095, *cysC*, *cysD*, OG098, *xcpT*, *xpsE*, *epsF*, *OG102*, *OG103*, *OG104*, *gspK*, *OG106*, *OG107*, *OG108*, *xpsD*, *bdbD*, and *wecA* (19 genes in total); IE2 contains *Rv2957*, *OG114*, *OG115*, *OG116*, *OG117*, *wbbL*, *OG118*, *OG119*, and *Rv2957* (9 genes); IE3 contains *gnu*, *espL*, and *pglF* (3 genes); IE4 contains *gmd*, *fcl*, *gmm*, *mand*, *glmM*, and *manA* (6 genes); IE5 contains *OG048*, *OG049*, *OG050*, *OG051*, *OG052*, *OG053*, and *OG054* (7 genes). Pandemic K-serogroups are indicated by star (*) according to a previous study (Han et al., 2016). Nonsense mutations of *rjg* and *gtaB* are indicated as yellow triangle and inverted triangle.

For the remaining 207 genes, 78% of them are distributed in fewer than three K serotypes (Supplementary Table 2 and Figure 4) and are mostly located in the middle regions of the CPSgc (Figure 3), responsible for the high diversity of CPSgcs (Pang et al., 2019). Average within-gene identity is 34.8%, which is less than those in core genes. Moreover, 16 genes have multicopies in several K serotypes (Figure 2, Table 3, and Supplementary Figure 4). The diversity of genes and position in the middle region are associated with diversity of the CPS loci in *V. parahaemolyticus*. This is consistent with the CPSgc characteristic among *Escherichia coli* K1, K4, and K5; *Neisseria meningitidis* serogroup B; and *Pasteurella multocida* types A, D, and F (Cress et al., 2014).

**TABLE 3:** Composition and frequency of multicopy genes in 40 K-serogroup CPSgcs.

Gene	Gene function class	Identity	Distribution frequency	Single-copy frequency	Dual-copy frequency	Tri-copy frequency	Mechanism*
*bshA*	Glycoltransferase	0.191	31	17	9	5	14/14 recombination
*Rv2957*	Glycoltransferase	0.184	30	13	15	2	2/17 (K3, K28) duplication; 15/17 recombination
*gtaB*	Pathway gene	0.348	29	23	3	2	5/6 recombination
*gmd*	Pathway gene	0.314	27	24	3	0	3/3 recombination
*pglF*	Pathway gene	0.509	22	17	5	0	5/5 recombination
*wecA*	Pathway gene	0.231	14	12	2	0	2/2 recombination
*fcl*	Pathway gene	0.466	14	13	1	0	1/1 recombination
*epsM*	Pathway gene	0.201	12	8	3	1	4/4 recombination
*desV*	Pathway gene	0.269	10	8	2	0	2/2 recombination
*wecB*	Pathway gene	0.215	7	6	1	0	1/1 duplication
*oatA*	Pathway gene	0.187	6	5	1	0	1/1 recombination
*epsG*	Processing and transportation genes	0.214	6	4	2	0	1/1 duplication
*wecC*	Pathway gene	0.168	5	4	1	0	1/1 duplication
*kdsB*	Pathway gene	0.176	4	3	1	0	1/1 duplication
*hepC*	Pathway gene	0.189	3	2	1	0	1/1 duplication

Column with * shows the mechanism of multicopy gene formation in terms of composition.

### Functional Characteristics of Genes in CPSgc

Pathway gene classes, containing 48 genes, are the largest of the three gene function classes (Table 2), and are mainly located in the middle and 3′ flank region of CPSgcs (Figure 3). Five pathway genes (*glpX*, *hldD*, *hpcD*, *tpiA*, *ugd*) are distributed in all 40 K-serogroups and the sequences of these five core genes are highly conserved with a mean within-gene pairwise identity of 83.9%, which is relatively high compared with any other genes (Table 2, Figure 2, and Supplementary Table 2), suggesting fundamental importance in CPS synthesis. For example, *ugd* encoding UDP-glucose dehydrogenase is putatively responsible for UDP-D-GlcA synthesis (Grangeasse et al., 2003; Cress et al., 2014). *gtaB* (also designated as *rmlA*) encoding UTP-glucose-1-puridylyltransferase and *glmM* encoding phosphoglucosamine mutase exist in 72.5 and 42.5% of 40 K-serogroups (Supplementary Table 4), respectively, and putatively participate in production of two CPS precursors, UDP-glucose and UDP-*N*-acetylglucosamine, respectively, as reported in many pathogenic bacteria (Cress et al., 2014). Three other genes, *rmlD*, *rfbC*, and *Fcl*, which are putatively involved in GDP-L-Fuc synthesis (Kneidinger et al., 2001) and frequently co-occur with gmd gene, and manC1, *glmM* and manA/manCBA, collectively encode proteins responsible for GDP-Man synthesis (Perepelov et al., 2015). These were identified to be especially conserved in insertion sequence 4 (see Section Group 5). *neuA* encoding CMP-*N*-acetylneuraminate cytidylyl transferase, which is putatively involved in polysialic acid biosynthesis in *E. coli* K1, is distributed in 17.5% K-serogroups (Supplementary Table 4). Interestingly, *wecB* (7/40) encoding UDP-*N*-acetylglucosamine-2-epimerase and *wecC* (5/40), which putatively catalyzes the formation of UDP-*D*-ManNAc (Campbell et al., 2000) and UDP-*D*-GalNAc (Cunneen et al., 2013), respectively, was found in several K serogroups, in particular group 3. In summary, the core pathway genes of 40 K-serogroups might be essential for synthesis of the common precursor of CPS, whereas the pan pathway genes may be catalyzed by the common precursor to a different product, which will change the chemical properties and immunogenicity of CPS.

Processing and transportation genes are responsible for forming the CPS repeat units and translocating mature CPS to cell surface subsequently (Tang et al., 2014). In total, 12 genes were identified to be processing and transportation genes class, and mainly located at the 5′ flank region of CPSgcs. Among them, two core genes (*kpsD* and *wzc*) were identified: *kpsD* gene, encoding polysialic acid transport protein (UniProt ID: Q03961), exists in all 40 K-serogroups with higher sequence identity 79.2%, whereas *wzc* gene, encoding tyrosine-protein kinase (UniProt ID: P76387), exists in all 40 K-serogroups with high sequence identity, except K28 and K3 with a low sequence identity of 31.5% (Supplementary Table 2 and Figure 2). *kpsD* is involved in translocation of polysialic acid capsule from the outer membrane to the cell surface, and *wzc* is possibly involved in the export of colanic acid from the cytosol to the outer membrane. Thus, we speculated that *kpsD* and *wzc* can non-specifically process and transport very distinct CPS structures. As illustrated in Section “Genetic Structural and Function Characteristics of CPSgc,” both of them are putatively integral to the CPS assembly along with other genes. Another processing and transportation gene, *epsL*, with high prevalence (29/40) is located among the pathway genes adjacent to the middle or 3′ flank of CPSgcs, together with *epsG* (6/40) in four K-serogroups. *EspL* reportedly plays essential roles in bacterial virulence due to type VII secretion system (Pearson et al., 2017; Sala et al., 2018), suggesting *epsL* might play multi-roles besides CPS biosynthesis. According to a recent study by Rendueles et al. (2017), CPS can be biosynthesized by using one of the following five mechanisms recognized by processing and transportation genes: Wzx/Wzy-dependent, ABC-dependent, synthase-dependent, PGA, and group IV. In our study, K28 and K3 belong to an ABC-dependent mechanism by our annotation strategy, whereas, unlike recognized as Wzx/Wzy-dependent mechanisms in Pang et al. (2019), other 38 K-serogroups cannot be identified in our study including K6 consistent with Chen et al. (2010).

Six genes classified into glycoltransferase genes class are mainly located in the middle region of CPSgcs (in orange in Figure 3) with frequencies ranging from 5 to 77.5% in 40 K-serogroups (Supplementary Table 2). These genes display lower mean pairwise identity within genes (average 21.5%) than other genes (Table 2 and Supplementary Table 2), such as *Rv2957* (as *galE* in Pang et al., 2019), putatively responsible for UDP-D-Gal synthesis (Samuel and Reeves, 2003), which displays high frequency at 30/40. Diversity and uniqueness among K-serogroups of glycoltransferase genes may determine the position and type of glycosyl in how polysaccharides occur differently in combination, which in turn gives rise to diversity of CPS (Mazmanian and Kasper, 2006).

### Evolution and Groups of 40 K-Serogroup CPSgcs

To investigate the evolutionary relationship of 40 K-serogroups, a species tree was established by using Orthofinder (Figure 4) and was rooted at K28 and K3 according to the phylogenetic relationship of core genes (Supplementary Figures 7A,B) and comparative analysis of CPSgc genetic structure (Figure 3), and its robustness were supported by other ML methods (Supplementary Figure 7A). We found that frequent recombination takes place via sequence insertion during the *V. parahaemolyticus* CPS locus evolution, consistent with previous findings in other pathogenic bacteria (Mostowy et al., 2014; Croucher et al., 2015; Wyres et al., 2015; Rendueles et al., 2017). Based on the phylogenetic relationship and genetic structure, 40 K-serogroups can be divided into five groups (groups 1–5) with each being characterized by certain insertion events and share more similar genetic structure than with K-serogroups in other groups (Figure 4).

#### Group 1

CPS loci of K28/K3 are much longer and possess more ORFs than do other K-serogroups (Figure 1B). They share similar structures and gene composition in proportion except for the 34th–41st and 34th–37th genes from the 5′ flank (Figure 3 and Supplementary Figure 3), but are very different from other K-serogroups. In species tree, K28 and K3 are clustered in an independent clade, designated as group 1, distinct from others both in core-gene tree (Supplementary Figure 7) and species tree (Figure 4). Thus, we put the root of the K-serogroup species tree between group 1 and the other K-serogroups. We proposed that during the evolutionary process, K28 and K3 were generated by two insertion events, namely, insertion events 1 and 2 (IE1, 2), from their ancestor, which putatively have similar genetic structure with other K serogroups (Figures 3, 4). Both 5′ and 3′ termini of IE1 sequence were featured by the gene *wecA*, whereas both 5′ and 3′ of IE2 sequence are terminated by *Rv2957* inversely (Figure 3). The genes in these two insertion sequences are uniquely distributed within group 1, supporting the notion that they were recombined from other species. However, it was not possible to identify the donor sources of these genes by homology searching in GenBank.

#### Group 2

Group 2 including K19, K32, and K17 are adjacent in the species tree and form a distinct clade in the core-gene tree at the root (Supplementary Figures 7A,B). An important structure feature is that the old border gene *rjg* locates at the middle of group 2 CPSgcs whereas it locates at the 3′ flank or absent in other groups. In addition, there is a group-specific gene *OG066* at the 3′ flank in group 2.

#### Group 3

Group 3 contains five K serogroups, namely, K5, K6, K58, K20, and K42, sharing similar CPSgc lengths ranging from 30 to 40 kb. This group differentiated from group 2 at node N1, and developed into group 4 and group 5 later (Figure 4). O3:K58 is a pandemic serotype identified in the post-pandemic period (Gavilan et al., 2013). The genetic structure and gene content of K6 and K58 CPS loci are very similar. The major difference is that *gtaB* (UTP-glucose-1-phosphate uridylyltransferase) is considered a pseudogene in K58 due to the presence of a nonsense mutation, suggesting that K58 may have originated from K6 (Figure 3).

#### Group 4

We proposed that another insertion event, IE3, has occurred in the ancestor (N10) of group 4 and group 5 (labeled with bold orange lines in Figures 3, 4). This insertion sequence specifically exists in group 4 (7/17) and group 5 (7/13) but not in other groups. The sequence of IE3 contains three genes *gnu*, *epsL*, and *pglF* with conserved order in 14 K-serogroups, frequently locating between the upstream genes *Rv2957* (10/14) or *bshA* (3/14), and the downstream core gene *ugd* (14/14). Blastn search against GenBank found that two hits from *Vibrio alginolyticus* have 98, 100% coverage and 91.7, 91.3% identity, which are presumably the potential donors of insertion event 3 sequence. In addition, this sequence is found in *Vibrio campbellii*, *Vibrio neocaledonicus*, and *Vibrio owensii* (Supplementary Table 3 and Supplementary Figure 6), indicating that the insertion sequence was potentially transferred among *Vibrio* species.

Another more recent insertion event, IE5, has occurred in K60, K68, K36, and K25, locating between an old border gene *rjg* and *tpiA* and destroying 5′ part of *rig* (labeled with bold red lines in Figures 3, 4). This is consistent with the insertion sequence in the 3′ junction region of K68 and K25 CPSgcs found by Masatoshi Okura et al., and 3 genes in the 3′ flank of IE5, *OG052*, *OG053*, and *OG054*, were annotated as transposase genes. We found that the insertion sequences of K68, K36, and K25 were potentially descending from the same ancestor, whereas in K60, it might descend from another donor source. The insertion sequence in K68, K36, and K25 contains seven genes (*OG048*, *OG049*, *OG050*, *OG051*, *OG052*, *OG053*, *OG054*), whereas in K60, it lacks gene *OG052* (Figure 3). The average identity of insertion sequence in K68, K36, and K25 is 99.9%, with only four nucleotide variations in the 7,234-bp sequence (data not shown). Blastn search against GenBank shows partial coverage hits for some of the genes against several *Vibrio* species, including *Vibrio splendidus* and *Vibrio cholerae* (Supplementary Table 3 and Supplementary Figure 6).

#### Group 5

Thirteen K-serogroups forming an independent clade were designated as group 5 (Figure 4). We inferred that the co-ancestor of these K serogroups underwent an insertion event, IE4 (at node N17), which makes the descendant CPSgcs share a six-gene sequence specifically within this group (labeled with blue bold lines in Figures 3, 4). The IE4 sequence contains six conserved genes with a fixed order (Figure 3), and exists in group 5 with high frequency (10/13 in this group, mean pair-wise identity 70%), but with low frequencies in group 3 (1/5) and group 4 (2/17). We speculate that the insertion sequence might have originated from an insertion event between the ancestral receptor *V. parahaemolyticus* K-serogroup and the donor species, and remained in group 5 during evolution, whereas a gradual loss has taken place in some K-serogroups. Blastn reveals that *Vibrio alginolyticus* (99% coverage, 96.3% identity) and *Vibrio harveyi* (89% coverage, 96% identity) are the potential donor species. Part of this sequence also was found in *Vibrio alfacsensis* and *Vibrio campbellii* (Supplementary Table 3 and Supplementary Figure 6).

These insertions are conserved within K-serogroups but are not strain specific. For example, insertion events 1 and 2 are distributed in all of 13 K28 and 13 K3 strains with an average identity of more than 99% and coverage average of 100%. These well-characterized insertion events mainly occur in the middle regions of CPSgcs, suggestive of high recombination activity.

### Gene Duplication Driving Evolution of *V. parahaemolyticus* CPS Loci

Multicopying of gene is a mechanism underpinning the generation of new genes and protein functional diversity (Haldane, 1932; Muller, 1935; Ohno, 1970). In 40 K-serogroups, we identified 16 multicopy genes, most of which were pathway genes (12), whereas others include 2 glycoltransferase genes, 1 processing and transportation gene, and 1 unclassified gene. Two pathway genes *gtaB* and *epsM* with triple copies exist at frequencies of 2/40 and 1/40, respectively (Table 3 and Figures 2, 3). Most multicopy genes occur less frequently than five K-serogroups. Such duplications appear conserved within K-serogroups. For instance, *bshA* duplication in K11 (7 strains), K56 (24 strains), and K8 (30 strains), *kdsB* duplication in K18 (3 strains), and *wecC* duplication in K17 (13 strains) are distributed in all corresponding K-serogroup strains with a high level of mean pairwise sequence identity (>95%). Remarkably, among these genes, the mean pairwise identities are negatively correlated with the frequency of multicopy (*r* = 0.5748), especially for genes distributed in more than 10 K serogroups (*r* = 0.8726). This means that the lower the mean pairwise identity, the higher the frequency of multicopy, supporting multicopy as a mechanism for gene divergence (Table 3).

We also found that the early evolution of CPSgc of *V. parahaemolyticus* may be promoted by frequent multicopying events. There is a total of 35 gene multicopying events in the process of CPS evolution, whereas most of them (71%, 25/35) are located at nodes N0–N4 (N0: 11 events, N1: 9 events, N2: 2 events, N4: 3 events) (Table 4 and Supplementary Table 4). Among these events, multicopying of two glycoltransferase genes, *Rv2957* and *bshA*, were most active, with 8 and 7 multicopying events, respectively. Notably, multicopying of *Rv2957* occurred during the whole process of evolution in such hotspots as nodes N0, N3, N13, and N27 (Table 4 and Figure 4). In analysis on a gene-by-gene basis, the two glycoltransferase genes, *Rv2957* and *bshA*, display dual copies at high frequencies of 15/40 (15 in 40 K-serogroups) and 9/40, respectively, and even triple copies at substantial frequencies 5/40 and 2/40. Most of the insertion events occur adjacent to *Rv2957* or *bshA* (25/36), and several others to another glycotransferase gene *tuaH* (3/36). Multicopying of glycoltransferase genes, *Rv2957* and *bshA*, might play an important role in the evolution of *V. parahaemolyticus* CPS loci and are well fixed in larger amounts of K-serogroups. Collectively, genes of multicopies might play important roles in K-antigen diversification especially through multiple pathway genes that catalyze a common precursor to different products.

**TABLE 4:** Distribution of duplication events on species tree of 40 K-serogroups.

	Node	Duplication event	Total event
1	N0	bshA*4, gtaB*2, epsM*3, desV*1, wecB*1	11
2	N1	bshA*4, Rv2957*4, gmd*1	9
3	N4	bshA*1, TTHA0252*2	3
4	N2	Rv2957*1, wecA*1	2
5	K17	hepC*1, wecC*1	2
6	N12	pgIF*1	1
7	N13	Rv2957*1	1
8	N27	Rv2957*1	1
9	N6	gtaB*1	1
10	N8	fcl*1	1
11	N9	oatA*1	1
12	K11	*bshA*	1
13	K18	*kdsB*	1
Total		35	35

We found that these multicopy genes may have been generated by two different mechanisms in *V. parahaemolyticus*. Ten of such genes display multicopies linked to recombination, most of which are pathway genes. Interestingly, nearly all of the pathway genes with high distribution frequency achieved multicopy by recombination, whereas those with low distribution frequencies tend to be generated by duplication (Table 3). Five multicopy genes, including four pathway genes (*wecB*, *wecC*, *hepC*, and *kdsB*) and one glycoltransferase gene (*Rv2957*), are generated by self-duplication (Table 3 and Supplementary Figure 8). *wecB*, *wecC*, and *hepC* duplication uniquely occurs in K17 with sequence identities of 72.3, 74.1, and 41.9%, respectively, and *kdsB* duplication uniquely occurs in K18 with 45.1% identities (Table 3), suggesting these duplication events may contribute to the origin of K17 and K18. There are three duplication genes in K17, which has the longest CPSgc except K28 and K3 (Figure 1B and Supplementary Table 1). Located on the sequence of insertion event 1 in K28 and K3, self-duplicated *Rv2957* is speculated to occur in the ancestral CPS loci of K28 and K3 and diverged along separation of K28 and K3. In addition, the duplicated *Rv2957*, which has an opposite direction both in K28 and K3, is a border gene of insertion event 2 sequence. Thus, they may have been acquired from other species and have promoted the sequence insertion by recombination.

## Discussion

Our findings have provided new insights into the global evolution and diversity of CPSgc of *V. parahaemolyticus*. Notably, our analysis has revealed the CPSgcs of 40 K-serogroups along with the finding of a conserved 3′-border gene and the conserved flank region, and variability in the middle region. Furthermore, we have constructed an evolution model encompassing insertion by recombination, gene duplication to account for the divergence of CPS loci, which should be helpful for our understanding of *V. parahaemolyticus* evolution and epidemics.

### Discovery of a New 3′-Border Gene of CPSgcs

Previous studies have suggested that CPSgcs of *V. parahaemolyticus* are located between *gmhD* and *rjg* (Chen et al., 2010). Our study reveals that the 3′-border gene *rjg* was not conserved among K-serogroups. More specifically, CPSgcs from five K-serogroups, K41, K38, K18, K28, and K3, do not contain *rjg*. In another five K-serogroups, K20, K29, K8, K11, and K44, *rjg* has undergone nonsense mutations splitting into two separated genes. In another three K-serogroups, K17, K19, and K32, *rjg* is located in the middle region but not the flank region of CPSgc (Figure 3).

We identified the gene *glpX* downstream of *rjg* as an accurate 3′-border gene of *V. parahaemolyticus* CPSgc, which is conserved in all 40 K-serogroups. During our study, Pang et al. (2019) extracted 55 K-serogroup CPSgcs by old 3′-border gene *rjg*. As we can see, for K18, K31, K38, K41, and K60 which do not have *rjg* and K17, K19, and K32 whose *rjg* is located in the middle region of CPSgc, entire CPSgcs have not been extracted in Pang et al. (2019), thus naturally will influence the comprehensive structure and function studies. Meanwhile, Chen et al. extracted K6 CPSgc losing seven 3′-flank genes (Chen et al., 2010). In summary, the new 3′-border gene *glpX* we found in this study is helpful to build a stable method to extract the entire CPSgc of *V. parahaemolyticus* and benefit to following functional research and diagnosis method development.

### Evolution Model for CPS Loci Incorporating Phylogeny and Comparative Genetic Structure Analysis

Based on our evolution analysis in the Results section, we propose the following evolution model of CPS loci in 40 K serotypes. We speculate that all 40 K-serogroup gene clusters share a common ancestor, designated as group 0 for convenience, whose genetic structure is highly similar to K17. During evolutionary processes, some of group 0 differentiated into group 2, and others differentiated into group 1 by two insertion events, IE1 and IE2, with the duplicated gene *Rv2957*. In group 1, K28 and K3 differentiated recently, as most of their gene order and sequence are conserved with only few different genes in the middle region of gene cluster. In group 2, K17 and other two K serogroups K19 and K32 are differentiated by three gene duplication events and recombination (Figure 3). Subsequently, group 3 differentiated from group 2 at node N1. K6 which developed to pandemic strains are in group 3. As is evident from our data, divergence of K6 and K58 was caused by mutations of the gene *gtaB*. Group 4 differentiated from group 3 at node N9 by insertion event 3, which leads to most gene cluster of group 4 and its derivative group 5 sharing IE3 sequence specially. Notably, in group 4, insertion event 5 sequence inserted K60, K68, K36, and K25 in parallel at a conserved position, which promoted these CPSgcs differentiating from others. Finally, group 5 differentiates from group 4 at node N15 by insertion event 4. This model represents the first step toward future systematic investigation on precise CPS genetic loci and CPS structure evolution. Our results also indicated that diverse pandemic K-serogroups capable of infecting humans have polyphyletically arisen along evolution of *V. parahaemolyticus* CPS loci. It is important to identify these genetic features and specific CPS structures to elucidate mechanisms of *V. parahaemolyticus* infection.

### Evolution Mechanism of *V. parahaemolyticus* CPS Loci

For the mechanism of variation and evolution of CPS’s structure and function, abundant literature shows that genetic variability in capsules can evolve rapidly across species by homologous recombination and horizontal transfer (Mostowy et al., 2014; Croucher et al., 2015; Wyres et al., 2015; Rendueles et al., 2017). Elsewhere, studies have proposed that recombination might have occurred between different sister species in *Vibrio*, such as between *V. cholera* and *V. mimicus* and between *V. harveyi* and *V.* campbelli (Sawabe et al., 2007). In this study, we found that not only insertion recombination (insertion events in this study) but also gene duplication promotes the evolution of CPS loci in *V. parahaemolyticus.* Other *Vibrio* species are potentially the major genetic donors for inserting into CPSgc (Supplementary Table 3 and Supplementary Figure 6). It is worth noting that most of these potential donor species (*Vibrio alginolyticus*, *Vibrio campbellii*, *Vibrio harveyi*) are evolutionary related, clustering in the same clade, termed the Harveyi clade by multilocus sequence analysis in a previous study (Sawabe et al., 2007). *Vibrio alginolyticus*, *Vibrio campbellii*, and *Vibrio harvey* have similar habitats as *V. parahaemolyticus* in seawater and some seafood (Molitoris et al., 1985; Thompson et al., 2007). Their growth on the chitinous exoskeletons of crustaceans can induce natural transformation in *Vibrionaceae* members (Sun et al., 2013). Collectively, these suggest that recombination with neighboring *Vibrio* species living in the same ecological niches might have promoted CPSgc evolution in *V. parahaemolyticus*. The study and surveillance of the emergence of the novel K serotype by natural transformation between *V. parahaemolyticus* and other closed *Vibrio* species deserve attention. In this study, we found that five multicopy genes have generated through gene duplication in some or all K-serogroups. Most of them belong to pathway gene class (4/5 pathway genes, 1/5 glycoltransferase genes), distributed in K18, K17, K28, and K3.

Interestingly, the old 3′-border gene *rjg* (in five K serotypes belonging to groups 3, 4, and 5) have simultaneously undergone nonsense mutations at different codons, while their downstream regions have undergone mutations leading to a start codon, thereby generating two different genes from a single ancestral gene *rjg* (Figure 4). A similar phenomenon was found in K58, where the gene *gtaB* has split to two neighboring genes by nonsense mutation, which is the only coding difference with respect to K6 (in group 3). The roles of nonsense mutation generating new genes in K-serogroup evolution might be another mechanism warranting further investigation.

### Origin of O3:K6 Serovariants

Most pandemic O3:K6 serovariants since 1996 were found to have gone through CPS loci recombination. The development of reliable methods for rapid identification of O3:K6 isolates (Matsumoto et al., 2000) has led to the serendipitous findings of other serotypes, such as O4:K68, O1:K25, and O1:KUT (untypeable), which contain *toxRS* sequences, AP-PCR profiles, ribotypes, and PFGE profiles identical to those of the O3:K6 serotype (Chowdhury et al., 2000a, b; Matsumoto et al., 2000). In addition to O4:K68, O1:K25, and O1:KUT, O6:K1, which share high molecular identity with an O3:K6 isolate, were detected in Taiwan (Wong et al., 2005). Therefore, these serotypes are regarded as “serovariants” of pandemic O3:K6 (Matsumoto et al., 2000). Subsequently, some studies have proposed molecular mechanisms for the conversion from pandemic O3:K6 to its serovariants. Through whole-genome comparisons, Chen et al. (2011) inferred that a recombination event involving a large region of 141 kb in length covering the O-antigen and K-antigen loci occurred in pandemic O3:K6 and gave rise to the new O4:K68 serotype. However, Han et al. (2008) postulated that the pandemic O4:K68, O1:K25, and O6:K18 post-1996 originated from pandemic O3:K6 by deletion or horizontal transfer of relevant O/K antigen genes.

In contrast, based on new evidence in our study, K6, K68, K25, and K18 are identified to belong to different groups with K6 in group 3, K68 and K25 in group 4, and K18 in group 5, and possess different genetic structures. As a result, the latest common ancestors of CPS loci for K68, K25, and K18 are presumably different from that of K6 (Figure 4). This means that K68, K25, and K18 could not have stemmed from K6 by gain or loss of genes. Instead, it is more reasonable to assume that homologous recombination of whole CPS loci between pandemic O3:K6 and non-pandemic environmental strains is a mechanism underlying the origin of pandemic O4:K68, O1:K25, and O6:K18. A similar phenomenon has been recognized as serotype switching in *Streptococcus pneumoniae* (Geno et al., 2015). On the other hand, our study also shows that pandemics novel K type may also arise through variations of CPS loci of pandemic O3:K6, such as pandemics O3:K58, and likely has originated from O3:K6 through gene nonsense mutation. This may be another mechanism for the evolution of novel serotypes/K types, which deserves attention in future investigation.

## Data Availability Statement

The whole genome assembles produced in this study have been deposited at DDBJ/ENA/GenBank under the bioproject accession PRJNA677930, and see BioSample Accession for each strain in Supplementary Table 1. Data on capsule biosynthesis loci sequence assembly generated in this study have been deposited in the publicly available CNSA database (https://db.cngb.org/cnsa/) under the project number CNP0000343 and in GenBank with accessions MT898002–MT898419 as listed in Supplementary Table 1.

## Author Contributions

LL, SB, and QH were involved in the conceptualization. SB, QL, YL, N-KW, MJ, LZ, and LL were involved in the data curation. SB, WZ, QL, and LL were involved in the formal analysis. QH and LL were involved in the funding acquisition. SB and LL were involved in the visualization and writing—original draft. SB, LL, WZ, and N-KW were involved in writing—review and editing. All authors contributed to the article and approved the submitted version.

## Conflict of Interest

The authors declare that the research was conducted in the absence of any commercial or financial relationships that could be construed as a potential conflict of interest.

## Footnotes

1 https://github.com/tseemann/shovill

## Supplementary Material

Supplementary Figure 1 Flow chart of CPSgc gene function confirmation.

Supplementary Figure 2 Functional contents of 40 K-serogroups displayed by proportion of four gene classes. The average percentage of pathway gene class in each K-serogroup is up to 39.48%: more specifically, K17 has the largest (52%) while K36 has the least pathway genes percentage (21.9%). The average percentage of processing and transportation gene class in 40 K-serogroups is 9.23%: K13 and K31 have the most (11.8%) while K60 has the least processing and transportation gene percentage (5%). The average proportion of glycoltransferase genes in 40 K-serogroups is the least which is equal to 8.43% in three gene classes: K19 has the most (15.2%) whereas K63 has the least percentage (2.4%).

Supplementary Figure 3 Genetic structure of 40 K-serogroup CPSgcs with same gene aligned. Compared with Figure 4, this figure connects the same gene in neighboring K-serogroups and only shows the first occurred gene name whose function is known from top to bottom.

Supplementary Figure 4 Presence or absence heatmap of all capsular genes of 40 K-serogroups. Supplemental to Figure 2, this figure shows all 217 genes of 40 K-serogroups.

Supplementary Figure 5 Heatmap profile for gene diversity assessment by within-gene pairwise identity. Genes occurring in more than 20 K-serogroups are displayed and sorted by distribution frequency and gene function class. Each cell shows the pairwise identity of all ORFs from two gene groups.

Supplementary Figure 6 Blast result of 5 insertion events. This is the graphic summary of 5 insertion events searching on the NCBI whose e-value is equal to 0. In this picture, the top 3 coverage results which are from foreign species are labeled by ^∗^ with different colors.

Supplementary Figure 7 Phylogenetic relationship inference of CPSgcs using different methods. Based on the alignment of concatenated sequences of 12 CPSgc core gene, which are the same ones used for Figure 4, trees are inferred by using **(A)** Fasttree with default JTT + CAT model, by using **(B)** IQ-TREE with the GTR + F + R9 model, or **(C)** by using raxml-ng with LG + G4 model. **(D)** Phylogenetic tree of CPSgc was inferred by using Fasttree with default JTT + CAT model based on the alignment of concatenated sequences of 6 core genes with no recombination signals, namely *ugd*, *hpcD*, *tpiA*, OG011, OG012, OG013, and *glpX*. These inferences were done with 1,000 bootstrap replicates, and the K-serogroup tips are colored according to Figure 4.

Supplementary Figure 8 16 gene trees that contain multi-copies. These are the gene trees of 16 genes which contain multi-copies. In every tree, tip labels refer to K-serogroup and gene ID. What is more, the red font with red box indicates the multi-copies originated by gene-self duplication, the blue and orange font refer to multi-copies originated by gene nonsense mutation and recombination apparently.

Supplementary Table 1 443 *V. parahaemolyticus* strain information in this study. Note: Information and accession numbers for capsule biosynthesis loci sequence assembly deposited in the publicly available CNSA database (https://db.cngb.org/cnsa/) and in GenBank as listed, whereas corresponding information on CPSgcs for strains that entire CPS gene cluster could not be extracted are indicated “—”.

Supplementary Table 2 Classification, mean pairwise identity, and distribution frequency of genes from 40 K-serogroup CPSgcs.

Supplementary Table 3 Blast identification of source of insertion events. Note: blast hits are ordered by score and the hits from other *Vibrio* species are highlighted in yellow.

Supplementary Table 4 Details for CPSgc gene duplication events. Each row shows CPSgc gene duplication event. The column with ^∗^ shows the node location of duplication event in Figure 4.
