# Supplementary figures and images for: Genetic Structure, Function, and Evolution of Capsule Biosynthesis Loci in Vibrio parahaemolyticus
Source: Front Microbiol. 2021 Jan 11;11:546150. doi: 10.3389/fmicb.2020.546150 (PMC7829505; doi:10.3389/fmicb.2020.546150)

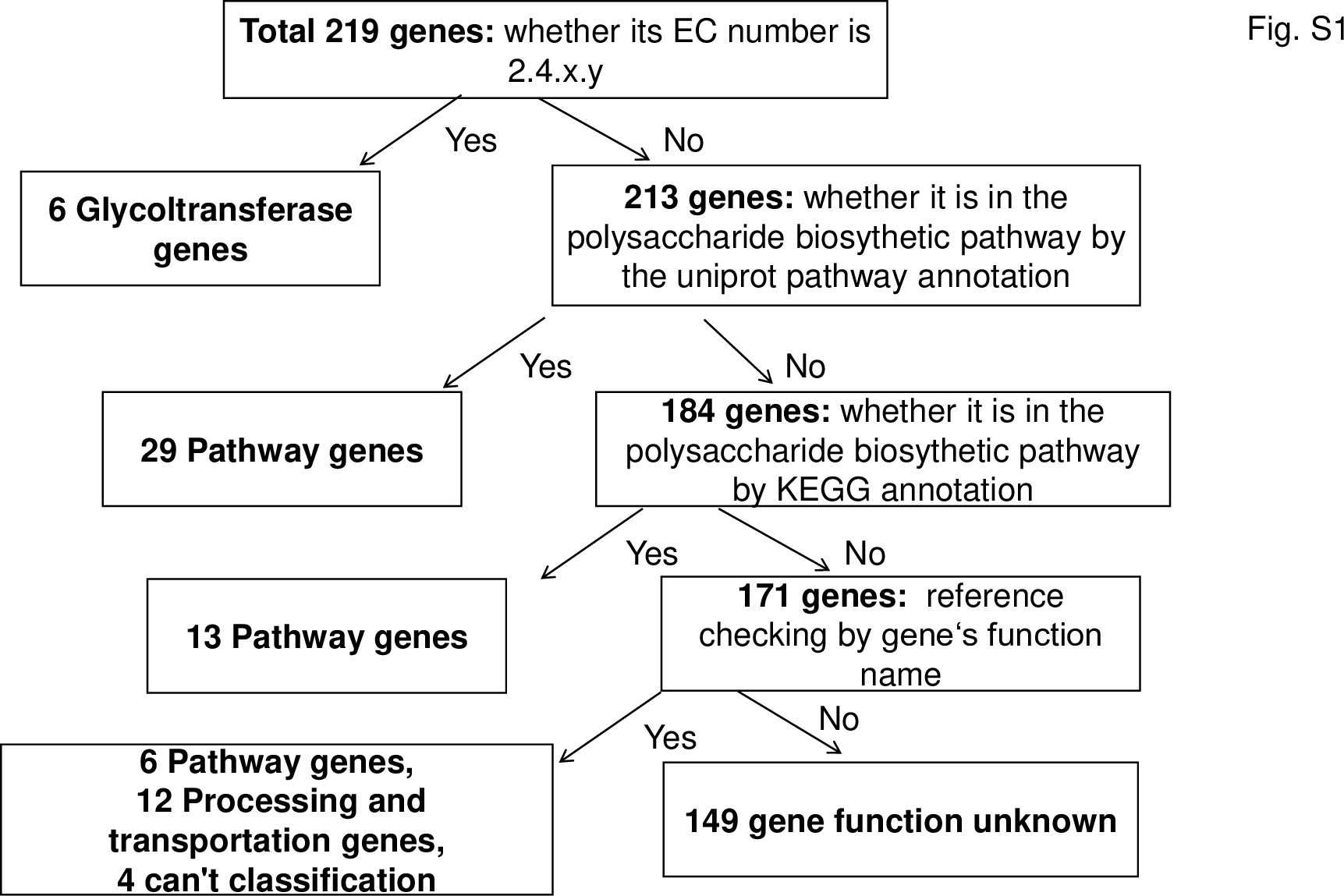

Supplement: Supplementary Figure 1 — Flow chart of CPSgc gene function confirmation. [file Image_1.TIF]

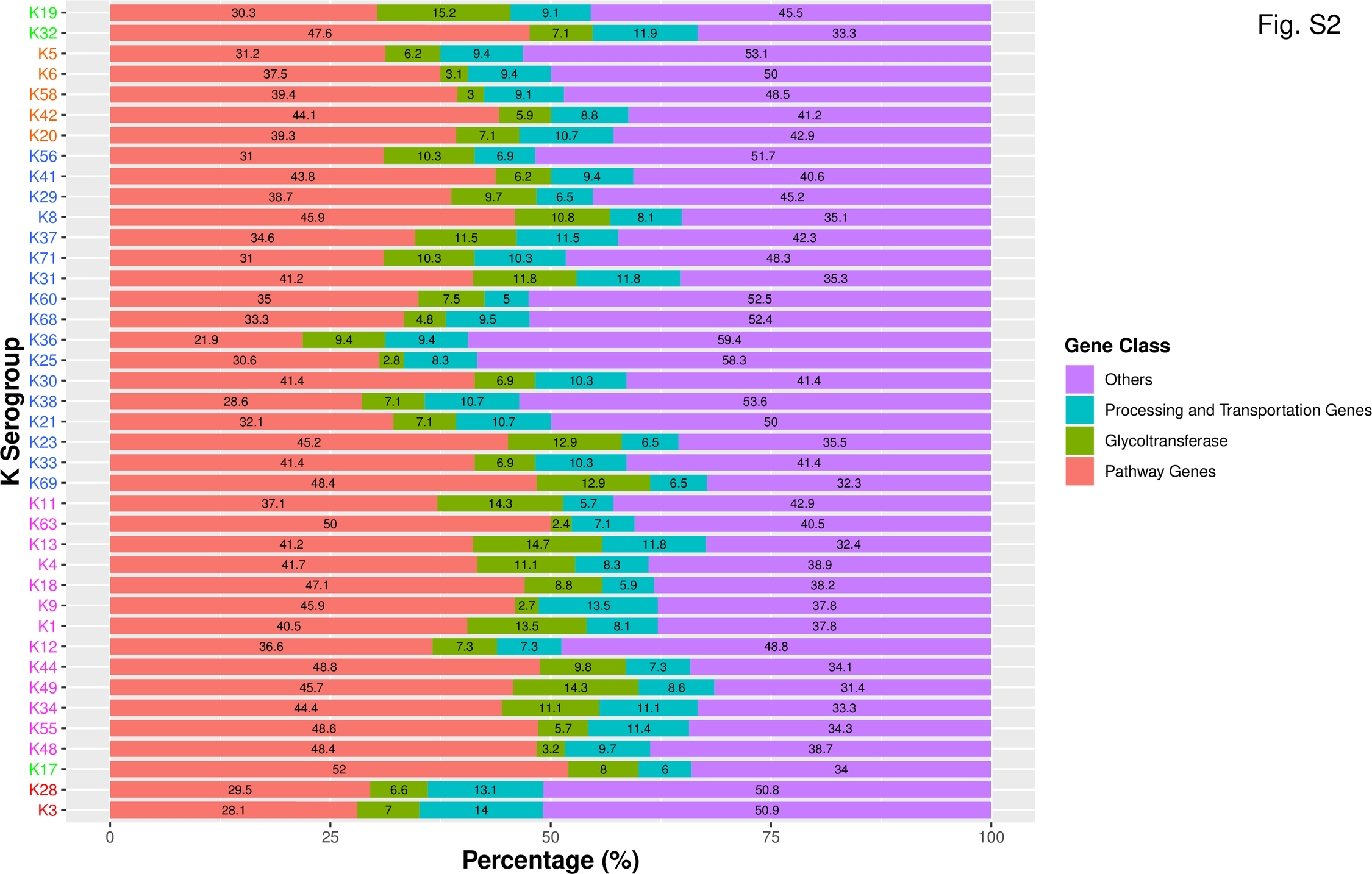

Supplement: Supplementary Figure 2 — Functional contents of 40 K-serogroups displayed by proportion of four gene classes. The average percentage of pathway gene class in each K-serogroup is up to 39.48%: more specifically, K17 has the largest (52%) while K36 has the least pathway genes percentage (21.9%). The average percentage of processing and transportation gene class in 40 K-serogroups is 9.23%: K13 and K31 have the most (11.8%) while K60 has the least processing and transportation gene percentage (5%). The average proportion of glycoltransferase genes in 40 K-serogroups is the least which is equal to 8.43% in three gene classes: K19 has the most (15.2%) whereas K63 has the least percentage (2.4%). [file Image_2.tif]

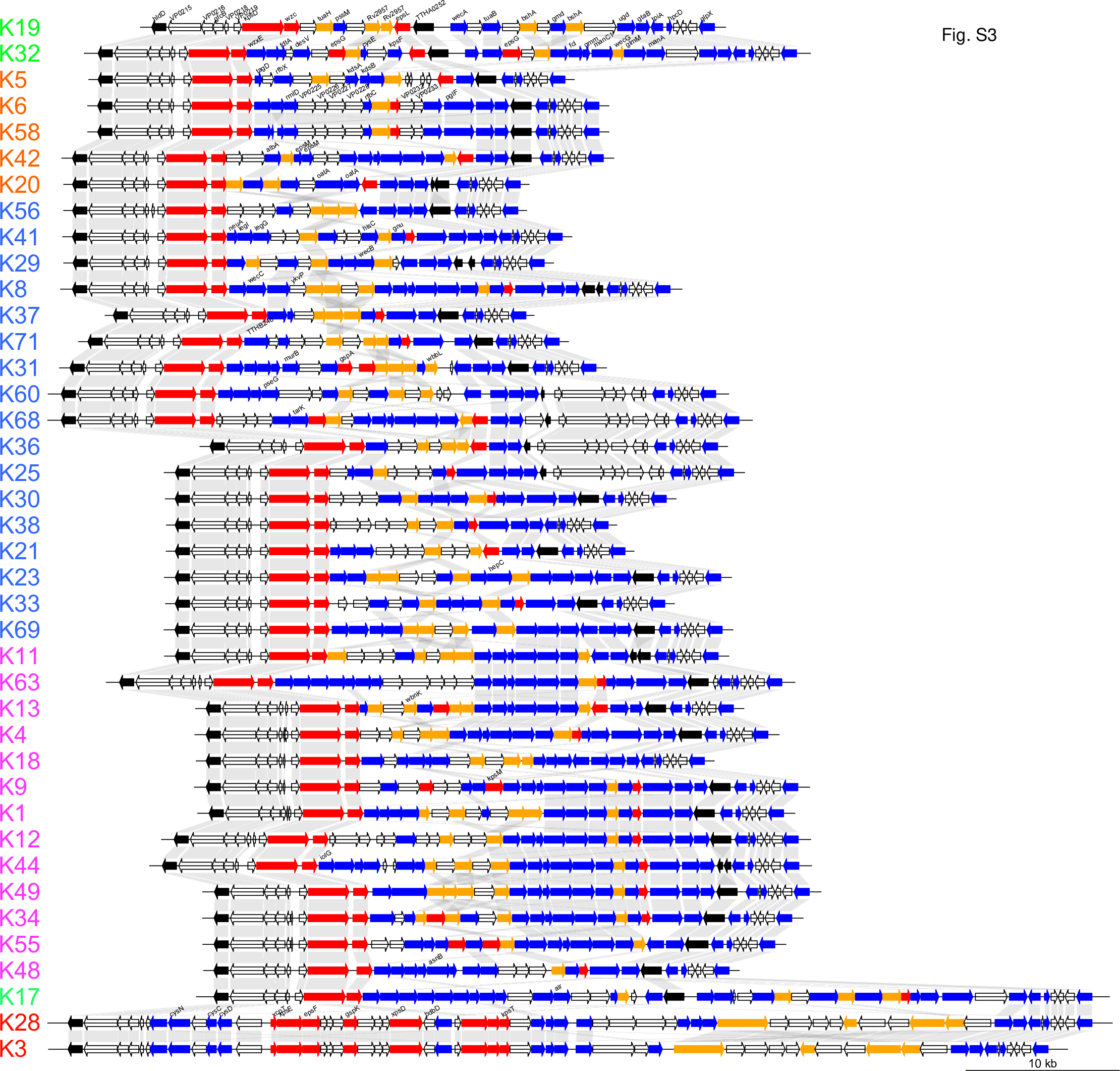

Supplement: Supplementary Figure 3 — Genetic structure of 40 K-serogroup CPSgcs with same gene aligned. Compared with Figure 4, this figure connects the same gene in neighboring K-serogroups and only shows the first occurred gene name whose function is known from top to bottom. [file Image_3.TIF]

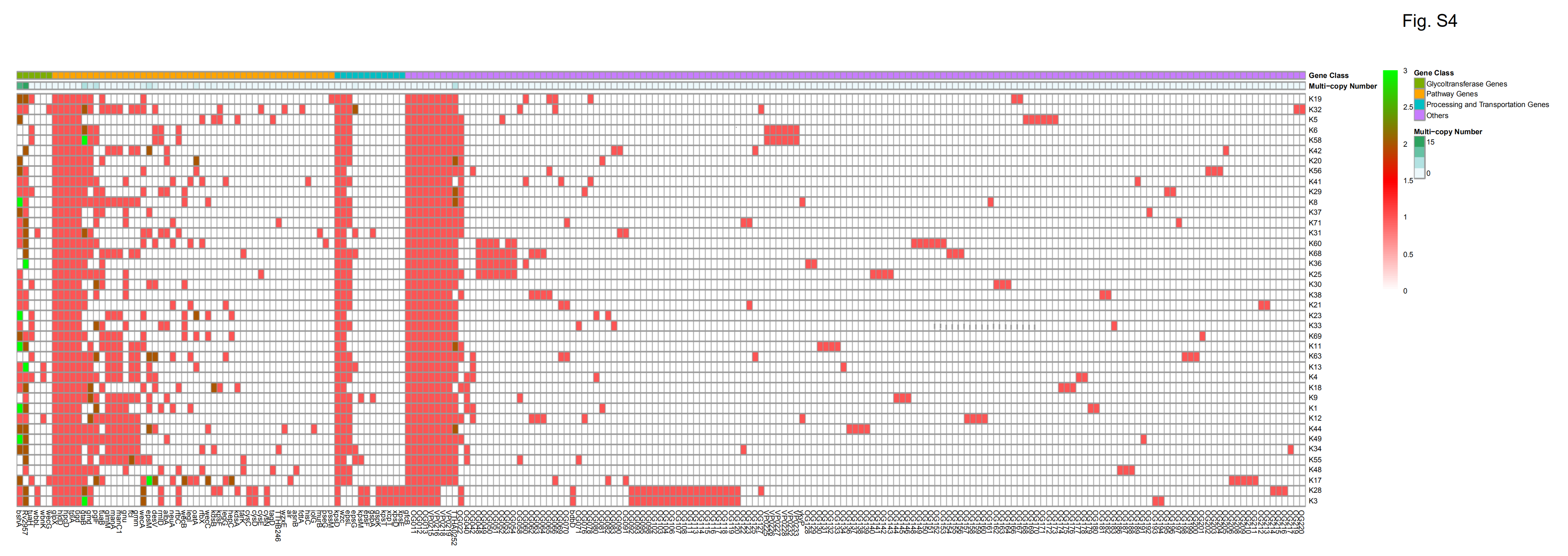

Supplement: Supplementary Figure 4 — Presence or absence heatmap of all capsular genes of 40 K-serogroups. Supplemental to Figure 2, this figure shows all 217 genes of 40 K-serogroups. [file Image_4.tif]

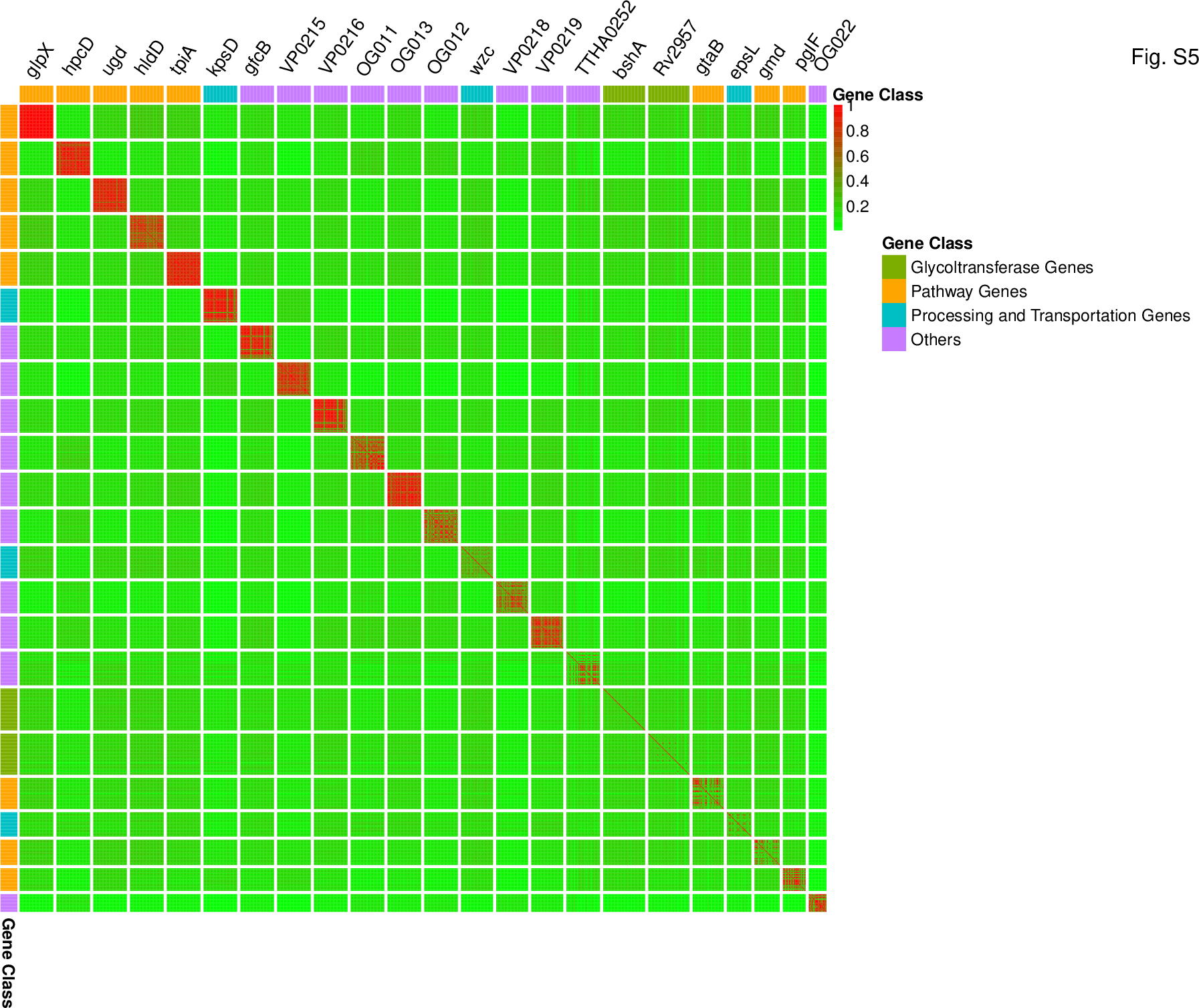

Supplement: Supplementary Figure 5 — Heatmap profile for gene diversity assessment by within-gene pairwise identity. Genes occurring in more than 20 K-serogroups are displayed and sorted by distribution frequency and gene function class. Each cell shows the pairwise identity of all ORFs from two gene groups. [file Image_5.TIF]

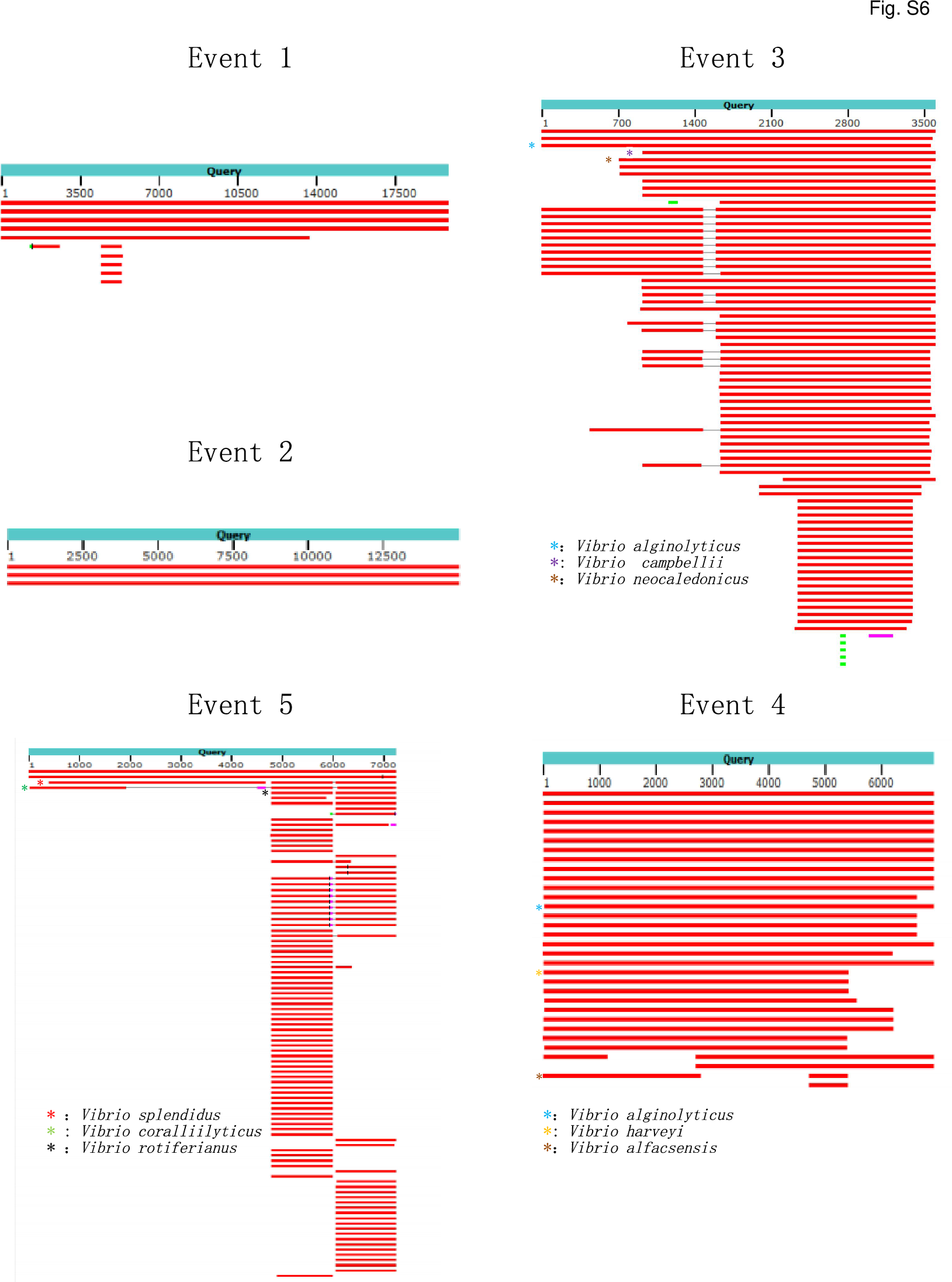

Supplement: Supplementary Figure 6 — Blast result of 5 insertion events. This is the graphic summary of 5 insertion events searching on the NCBI whose e-value is equal to 0. In this picture, the top 3 coverage results which are from foreign species are labeled by ∗ with different colors. [file Image_6.TIF]

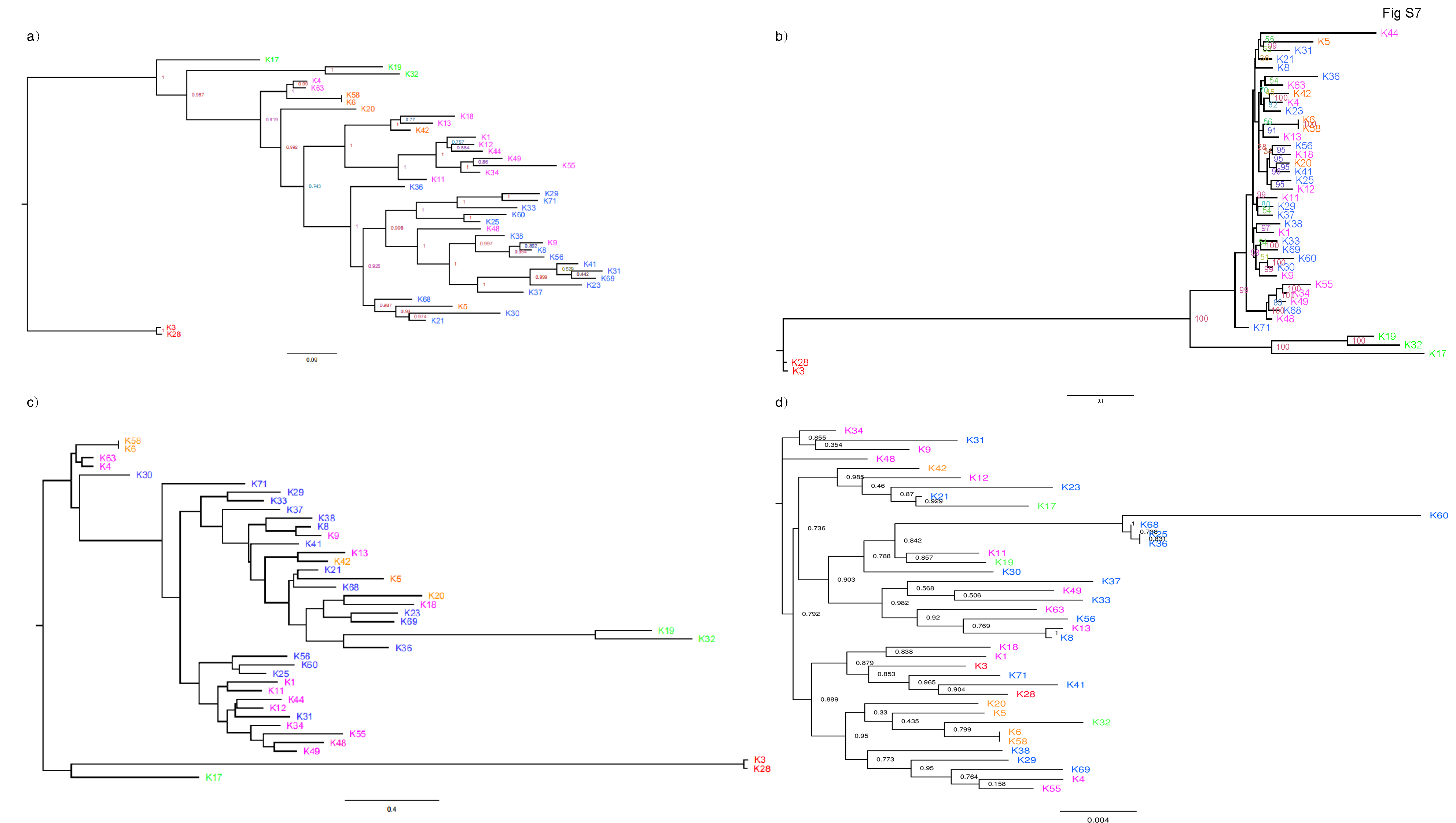

Supplement: Supplementary Figure 7 — Phylogenetic relationship inference of CPSgcs using different methods. Based on the alignment of concatenated sequences of 12 CPSgc core gene, which are the same ones used for Figure 4, trees are inferred by using (A) Fasttree with default JTT + CAT model, by using (B) IQ-TREE with the GTR + F + R9 model, or (C) by using raxml-ng with LG + G4 model. (D) Phylogenetic tree of CPSgc was inferred by using Fasttree with default JTT + CAT model based on the alignment of concatenated sequences of 6 core genes with no recombination signals, namely ugd, hpcD, tpiA, OG011, OG012, OG013, and glpX. These inferences were done with 1,000 bootstrap replicates, and the K-serogroup tips are colored according to Figure 4. [file Image_7.tiff]
