# Supplementary material for: Genetic Structure, Function, and Evolution of Capsule Biosynthesis Loci in Vibrio parahaemolyticus
Source: Front Microbiol. 2021 Jan 11;11:546150. doi: 10.3389/fmicb.2020.546150 (PMC7829505; doi:10.3389/fmicb.2020.546150)
Supplement: Supplementary Figure 8 — 16 gene trees that contain multi-copies. These are the gene trees of 16 genes which contain multi-copies. In every tree, tip labels refer to K-serogroup and gene ID. What is more, the red font with red box indicates the multi-copies originated by gene-self duplication, the blue and orange font refer to multi-copies originated by gene nonsense mutation and recombination apparently. [file Image_8.pdf]

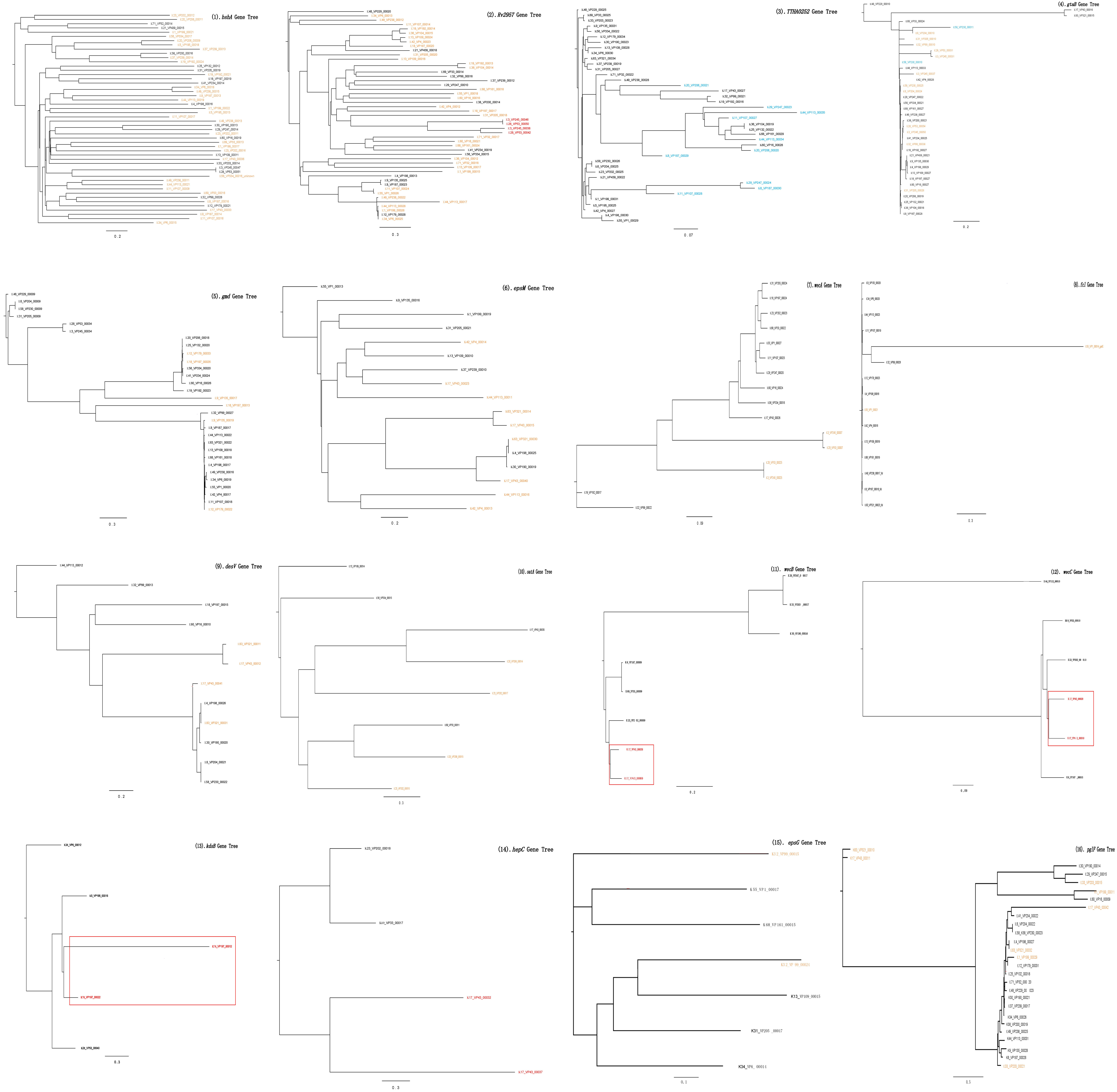
(1). bshA Gene Tree
(2). Rv2957 Gene Tree
(3). TTHA0252 Gene Tree
(4). gtaB Gene Tree
(5). gmd Gene Tree
(6). epsM Gene Tree
(7). wecA Gene Tree
(8). fcl Gene Tree
(9). desV Gene Tree
(10). oatA Gene Tree
(11). wecB Gene Tree
(12). wecC Gene Tree
(13). kdsB Gene Tree
(14). hepC Gene Tree
(15). epsG Gene Tree
(16). pglF Gene Tree
